# The Effects of Bioactive Compounds on Human Brain Structures and Diseases

**DOI:** 10.3390/ijms25063326

**Published:** 2024-03-15

**Authors:** Terezia Kiskova, Benadik Smajda

**Affiliations:** Department of Animal Physiology, Institute of Biology and Ecology, Faculty of Science, Pavol Jozef Safarik University, 041 54 Kosice, Slovakia; benadik.smajda@upjs.sk

The human brain is the most intricate organ in the body. Throughout its development, environmental influences shape its trajectory, for better or for worse [1]. Because the brain is a vulnerable structure, it may be damaged during development, as well as during adulthood.

Brain diseases are the leading cause of disability-adjusted life years (DALYs) and the second leading cause of death [2,3]. Studies on complex neurodevelopmental disorders like schizophrenia and autism have revealed that a specific clinical syndrome can be linked to a diverse range of genetic risk factors. This implies the existence of multiple pathways from genetic makeup to observable behavior [4,5,6].

The aim of this Special Issue is to spotlight the mechanisms through which various bioactive compounds can promote neuroprotection in the brain and potentially prevent or treat brain diseases, such as neurodegenerative disorders, ischemic attacks, brain cancer, or depression.

Attention in the field of drug discovery has increasingly been focused on neuroprotection using natural compounds from traditional medicinal herbs [7]. According to the WHO, 80% of the global population relies on medicinal plant-based medicine to mitigate or cure diseases [8]. Plants are a source of pharmacologically potent drug molecules of high efficacy. Recently, herbal medicine has undergone rapid evolution, garnering globally acceptance owing to its natural origin [5]. Traditional medicines could represent an alternative option to allopathic treatments for curing various neurodegenerative disorders, because the latter are limited and can have severe adverse effects [9]. Natural products, secondary metabolites, and bioactive molecules derived from plants and microorganisms are key sources of bioactive molecules [10].

Herbal products contain complicated mixtures of organic chemicals, which may include fatty acids, sterols, alkaloids, flavonoids, glycosides, saponins, tannins, terpenes, etc. [11]. Metabolites such as tannins, anthocyanins, and alkaloids serve as a defense mechanism in live organisms and are undoubtedly compounds of interest in the food, cosmetic, and pharmaceutical industries [12].

Supporters of herbal medicines assert that the therapeutic value of plants arises from the synergistic effects of their various components, in contrast to the isolated chemicals identified by pharmacologists in conventional medicines. Consequently, traditional medicines are believed to be effective and have minimal or no side effects [11]. Bioactive compounds derived from plants have garnered significant attention because of their firmly established medicinal properties, including anti-apoptotic, antioxidant, anti-inflammatory, anticancer, antimicrobial, neuroprotective, hepatoprotective, and cardioprotective, properties [13].

Extracts from various plants are commonly used in traditional medicine, either alone or in combination [11]. Traditional Chinese medicine (TCM) is based on the balance between health and disease. Herbal medicines with memory-enhancing properties are also encompassed in TCM. The plants used in TCM for memory improvement include *Huperzia serrata*, *Alpinia oxyphylla*, *Ginkgo biloba*, *Panax ginseng*, *Tripterygium wilfordii*, *Ganoderma lucidum*, *Camellia sinensis*, *Astragalus membranaceus*, *Polygonum multiflorum*, *Acanthopanax senticosus*, *Achyranthes bidentata*, and many others [7,14,15].

The wealth of new studies in this field underscores the undeniable potential of natural compounds and herbal mixtures, underscoring their crucial roles in molecular neuroprotective mechanisms. These findings may pave the way for the development of protective agents derived from traditional herbal medicine for addressing neurodegenerative and neurological disorders.
